# Airway remodeling: The *Drosophila* model permits a purely epithelial perspective

**DOI:** 10.3389/falgy.2022.876673

**Published:** 2022-09-15

**Authors:** Birte Ehrhardt, Natalia El-Merhie, Draginja Kovacevic, Juliana Schramm, Judith Bossen, Thomas Roeder, Susanne Krauss-Etschmann

**Affiliations:** ^1^ Division of Early Life Origins of Chronic Lung Diseases, Research Center Borstel, Airway Research Center North (ARCN), German Center for Lung Research (DZL), Borstel, Germany; ^2^ Division of Molecular Physiology, Institute of Zoology, Christian-Albrechts University Kiel, Kiel, Airway Research Center North (ARCN), German Center for Lung Research (DZL), Kiel, Germany; ^3^ Institute of Experimental Medicine, Christian-Albrechts-Universität zu Kiel, Kiel, Germany

**Keywords:** *Drosophila melanogaster*, airway epithelium, airway remodeling, animal model, chronic inflammatory lung diseases, trachea

## Abstract

Airway remodeling is an umbrella term for structural changes in the conducting airways that occur in chronic inflammatory lung diseases such as asthma or chronic obstructive pulmonary disease (COPD). The pathobiology of remodeling involves multiple mesenchymal and lymphoid cell types and finally leads to a variety of hardly reversible changes such as hyperplasia of goblet cells, thickening of the reticular basement membrane, deposition of collagen, peribronchial fibrosis, angiogenesis and hyperplasia of bronchial smooth muscle cells. In order to develop solutions for prevention or innovative therapies, these complex processes must be understood in detail which requires their deconstruction into individual building blocks. In the present manuscript we therefore focus on the role of the airway epithelium and introduce *Drosophila melanogaster* as a model. The simple architecture of the flies’ airways as well as the lack of adaptive immunity allows to focus exclusively on the importance of the epithelium for the remodeling processes. We will review and discuss genetic and environmentally induced changes in epithelial structures and molecular responses and propose an integrated framework of research for the future.

## Introduction

Airway wall remodeling is a complex pathology that occurs in different chronic inflammatory lung diseases such as asthma, chronic obstructive pulmonary disease (COPD), or bronchopulmonary dysplasia in preterm infants and is ill-responsive to treatment. These conditions involve lasting but variable changes in epithelial, airway smooth muscle and vascular cells, the subepithelial reticular basement membrane and deposition of extracellular matrix proteins each to different degrees. Airway remodeling has for long been thought to result from the long duration of the disease and concomitant chronic inflammation and therefore represent an advanced stage of pathology. This notion is now questioned as basal membrane thickening has been described already in infants and preschoolers with wheeze (1, 2) or childhood asthma (3).

Given the many cell types involved and the difficulty in reversing airway remodeling with therapeutics, the deconstruction of complex biological processes into cell-specific events could help to better understand the pathophysiology and find new targets for intervention. Following this reductionist approach, this review will focus on the airway epithelium which constitutes more than just an interface between the internal and the external environment. Epithelial cells serve as first line of defense against airborne pathogens, physical and chemical damage (4–6). Besides their function as cellular barrier, they provide mucociliary clearance of the lung and are involved in immune response by releasing pro- and anti-inflammatory mediators (6–9).

**Figure 1:**
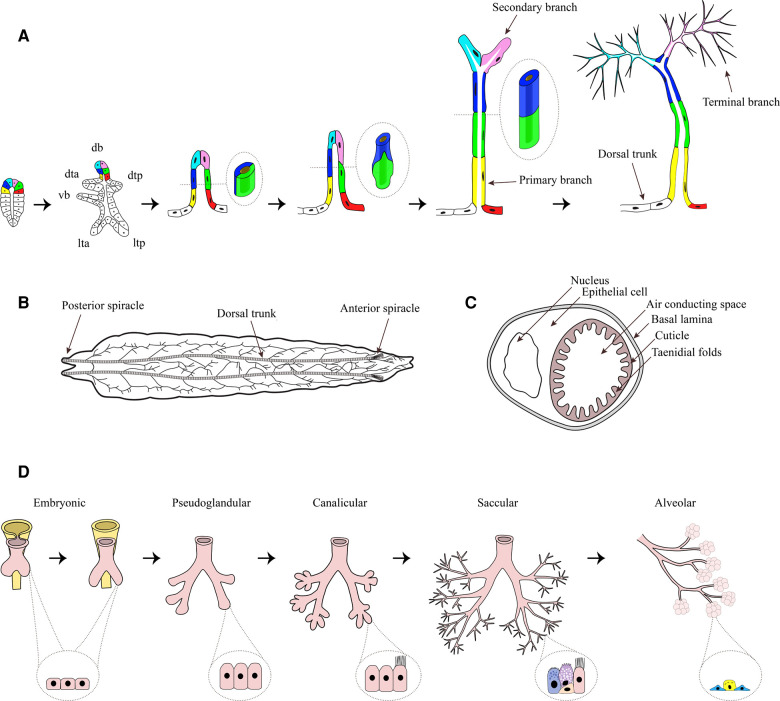
Tracheal system of *Drosophila melanogaster*. (**A**) Tracheal development begins with the formation of a tracheal sac in each segment (schematic drawing on the left). From the tracheal sac, six primary branches, namely dorsal branch (db), dorsal trunk anterior (dta), dorsal trunk posterior (dtp), visceral branch (vb), lateral trunk anterior (lta), and lateral trunk posterior (ltp) arise by migration of small groups of tracheal cells, which organize themselves into tubes. During ongoing primary branch formation the cells rearrange from an side-by-side to an end-to-end localization (shown in the enlargements). From the primary branches, secondary branches arise, which generate the fine terminal branches by growing of cytoplasmic extensions. Figure adapted from (60). (**B**) Dorsal view of an L3 larvae. The two dorsal trunks connect the anterior spiracle and the posterior spiracle, while primary branches are branching off in a stereotyped manner, building the typical tracheal ramifications. (**C**) Cross-section of a trachea. The tube is built of a single epithelial cell, surrounding the air conducting space. On the basal side of the cell a basal lamina can be found, while on the apical side a cuticle is secreted by the epithelium which forms taenidial folds, projecting into the lumen. (**D**) Stages of human lung development: Embryonic (formation of lung bud major airways, epithelium with progenitor cells), Pseudoglandular (formation of bronchioles, columnar cells), Canalicular (formation of distal airways and branching, differentiation of ciliated cell), Saccular (expansion of airspace, basal and goblet cells) and Alveolarization (alveolar cells type 1 and 2). Parts of the figure was created with BioRender.com.

Structurally, mammalian airways are lined by pseudostratified columnar ciliated epithelium which is composed of a variety of distinct cell types (Figure 1D), including basal cells, ciliated cells, club cells and goblet cells (10–13) and rarer populations such as tuft cells and ionocytes (14–16). Basal cells serve as progenitors which differentiate into other cell types, club cells secrete surfactants, goblet cells secrete mucous and together with the ciliated cells drive the mucociliary clearance (10, 11). In addition to these resident cells, a variety of other cells such as macrophages, mast cells, and dendritic cells, migrate and reside within the epithelium (17).

The maintenance of epithelial integrity is crucial for lung homeostasis and cell signaling, whereas the destruction of the epithelial barrier not only alters the normal function of epithelial cells but also has important implications for development and progression of chronic respiratory diseases such as COPD and asthma (4, 18, 19). Thus, alterations in the airway epithelial architecture and function are associated with transcriptional (20, 21) and epigenetic (22–24) reprogramming of epithelial cells (25–28). For instance, smoking induces transcriptional reprogramming of the airway epithelial apical junctional complex architecture thus disrupting epithelial barrier and increasing epithelial permeability (21). Moreover, smoking induces transcriptional upregulation of tuft-like cells and mucin producing cells and a decrease in ionocytes (29). These changes in gene regulation lead to goblet cells hyperplasia, reduce mucociliary clearance, trigger thickening of the basal lamina, smooth muscle hypertrophy and epithelial disruption (30, 31). Along with these changes, the epithelium becomes more permeable, allowing the entry of pathogens into the airway submucosa (31, 32). In order to restore homeostasis and barrier integrity, the respiratory epithelium initiates repair processes immediately after injury. However, repetitive injuries of the airway epithelium can finally compromise the normal repair mechanisms thus inducing inflammation associated with asthma and COPD (33, 34).

The exact mechanisms leading to such persistent epithelial changes are still unknown. Human data on dysregulated epithelial repair is observational and does not allow the study of the underlying mechanisms and the prospective clinical relevance (26, 35, 36). Therefore, animal models which will recapitulate the pathophysiology of the disease and hence allow the development of new therapy are highly instrumental. A variety of animal models, such as mice, rats, guinea pigs, sheep, and cats, have been introduced to reflect airway disruption, where mice are the most common mammals used (37–44). Alterations in lung development upon exposure to allergens like house dust mite (HDM) and ozone have been investigated in rhesus macaques, where alveoli number was not only significantly increased compared to filtered air exposed animals but also their capillary densities have been higher. This provided insights into early postnatal events in lung parenchyma very similar to humans (45). Chronic exposure to allergens like HDM and *Aspergillus fumigatus* are now used to mimic airway remodeling in the context of allergic airway inflammation in murine model (46–49). Investigating *A. fumigatus*-induced airway remodeling led to the assumption that the asthmatic milieu provides optimal growth conditions for the fungus, whereby from *A. fumigatus* produced allergens and metabolic by-products could possibly destroy the integrity of the epithelium during germination Mimicking airway remodeling in murine models therefore may provide insight into the interactions of this fungus with the epithelium, but cannot provide information about the function of individual cell types in the epithelium during fungal infection (49). To mimic not only airway but also vascular remodeling in asthma, mice were sensitized with ovalbumin (OVA) leading to development of an IgE-mediated hyper-reactive airway disease. Yet it remains unclear how individual cell types react to OVA treatment (50). Similarly, COPD mouse models have evolved where cigarette smoke or lipopolysaccharide (LPS) have been used to mimic airway inflammation and destruction (51–53). Although mouse models used have provided useful insights into the development of airway remodeling, a deeper understanding of the pathogenesis of airway remodeling in asthma and COPD is complicated by the heterogeneity of the tissue units forming the airways that are involved in this process (18, 26).

While the mammalian airway epithelium consists of different cell types such as goblet, multi-ciliated, club, basal and neuroendocrine cells, Drosophila airways are composed of a single layer of uniformly arranged epithelial cells, highlighting the simplicity of the model (54, 55). Therefore, to improve our insight into airway epithelial barrier dysfunction in the pathogenesis of asthma and COPD, it is important to study the normal function and architecture of solely the airway epithelium. Thus, a simple model such as fruit fly *Drosophila melanogaster* would be ideal to study molecular changes behind the pathogenesis leading to airway remodeling.

To understand and therefore enable the development of innovative solutions for prevention and therapies, the complex mechanisms contributing to disease development must be understood in detail. This requires dismantling of the complex processes into fundamental building blocks. Therefore, it might be advantageous to use less complex model systems such as the fruit fly *D. melanogaster*. The simplicity of this model is also evident in the immune system. Fruit flies, compared to more complex models such as mice or rats, lack an adaptive immune system. Therefore, their immune defense relies completely on innate immune reactions (55–57). Similar to humans, the epithelial cells of the fly form an important barrier to harmful environmental influences, which makes this model very attractive for studying the pathobiology of airway remodeling (55).

In the current review, we will therefore focus on the airway epithelium of *D. melanogaster* as a model system that exhibits a promising potential for studying the mechanisms implicated in airway remodeling. We will review and discuss genetic and environmentally induced changes in the epithelial structures as well as the molecular responses and propose an integrated framework of research for the future.

## The tracheal system of *Drosophila melanogaster* and its use in studying remodeling processes

The respiratory organ of the fruit fly *D. melanogaster* is the tracheal system, which is a network of epithelial tubules. This network transports gases from the respiratory openings, termed spiracles, which connect the system to the outside, to the tissues and organs *via* tracheal branches ramifying through the body.

The branching pattern of the tracheal system during the development is complex, but highly conserved and displays segmental repetition as well as bilateral symmetry (58). During the early embryonic development a tracheal sac forms in each segment by invagination of tracheal precursor cells (59) (Figure 1A). As the trachea is invaginated, the progenitor cells of the trachea undergo their final cell division, giving rise to the approximately 80 cells of the tracheal sac (59, 61). In each segment, the whole tracheal system is formed from these 80 cells, which means that no further cell division or cell death occurs (61). Thus, the tubular system is built by cell migration as well as changes in cell size, shape and intercellular contacts (59, 61). One of the main regulators of tracheal cell identity is the transcription factor Trachealess (*trh*), which is expressed in all tracheal cells (62, 63). Without *trh* tracheal development is severely compromised (62, 63) since *trh* controls a number of other tracheal genes, including *breathless* (*btl*) and *rhomboid* (*rho*) (62, 63). While *rho* is important for the activation of the epidermal growth factor (EGF) signaling pathway, which initiates local cell movements (64), *btl* encodes the homologue of mammalian fibroblast growth factor (FGF) receptor, which enables tracheal cell migration led by FGF signaling as chemoattractant (59). Recently it was shown, that impaired FGF- signaling is strongly associated with reduced growth (65). The knockdown of *btl* significantly reduced terminal branching as well as the total length of tracheae, resulting in hypoxic conditions of tissues. Further, the tracheal knockdown of *btl* led to an altered expression and reduced secretion of *Drosophila* insulin-like peptides 2, -3, and -5 from the insulin producing cells, which finally resulted in an insulin-driven reduced systemic growth rate (65). The data suggest the fat-body as the central organ for integration of hypoxia and amino-acid sensing as well as insulin regulation, thus supporting the concept of the tracheal system being part of a size-assessment mechanism. Similar mechanisms are operative in humans, as EGF was shown to be implicated in the development of airway remodeling of wheezing infants (66) and FGF signaling is linked to airway remodeling during early-life rhinovirus infection of children (67).

From the tracheal sac, six primary branches (Figure 1A) arise by migration of small groups of tracheal cells, which organize themselves into tubes (61). In a first step, these cells form tubes with a 2-cell diameter thereby forming primary branches (Figure 1A). In the second stage of primary branch formation, the cells elongate and rearrange from a side-by-side to an end-to-end localization (61) (Figure 1A), resulting in unicellular tubes (68). The primary branches that form the dorsal trunk do not undergo the second step but remain clustered, forming multicellular tubules (68), while they fuse with the branches in neighboring segments (61).

All other primary branches, not forming the dorsal trunk, give rise to secondary branches. Most secondary branches form at the end of primary branches, and only few arise at defined internal positions. Each secondary branch is formed by a single tracheal cell (61), generating a tube by building autocellular junctions along its length (59). These cells also shape the third type of tracheal tubes, the tracheal terminal branches or tracheoles, by growing cytoplasmic extensions along the surfaces of target tissues and subsequently forming intracellular lumen (59, 61, 68) (Figure 1A). The process of terminal branching continues during larval life, resulting in terminal cells with complex branched structures and dozens of terminal branches (59). The number of terminal branches is not limited but rather driven by oxygen availability (58). More terminal branches are produced under hypoxic conditions. It could be shown that long-term developmental response to hypoxia is at least partly induced by the activation of nitric oxide synthase and the production of nitric oxide (69). The sprouting of new terminal branches under hypoxia is mediated by *branchless* (*bnl*) FGF signaling (65, 70), which is induced by the accumulation of *Drosophila* HIF-α homolog *Sima* that leads to the enhanced expression of *btl* (71). Secondary and tertiary branching seem to be dependent on the expression of *hindsight* (*hnt*), a nuclear-zinc finger protein which possibly acts as a transcription factor to maintain epithelial integrity and initiate differentiation of the cuticle to form taenidial folds (72). While primary branching occurs normally, *hnt*-mutants show loss of epithelial organization accompanied by failure of secondary and tertiary branching, as well as loss of taenidial folds of the inner cuticle during the late embryogenesis, suggesting *hnt* to be a key factor for tracheal development during this developmental stage (72).

In the larvae the two dorsal trunks form the main tubes, connecting the anterior spiracle in the first thoracic segment to the posterior spiracle in abdominal segment 8 and thereby linking the metameric units (58) (Figure 1B). From the dorsal trunks primary branches are branching off in a stereotyped manner. Each branch migrates towards its target tissue (68). The inner primary branches are short and proceed dorsally, while the outer primary branches form a consistent lateral trunk, running a jagged course from segment to segment. Transverse connectives interconnect the dorsal and lateral trunk in each metamere (58).

During metamorphosis the tracheal system undergoes drastic reconstructions, to first form the pupal and then the adult tracheal system. Like in larvae, the adult tracheal system is stereotyped, with the fine terminal branches being variable in response to the requirements of target tissues (58).

Histologically each respiratory branch consists of one layer of epithelium encompassing the air conducting space. The oxygen is transported through the epithelium to reach target organs and tissues. On the basal side, the epithelium is covered by a basal lamina. The apical (luminal) surface of the epithelium is surrounded by an extracellular matrix, the cuticle, which is secreted by all epithelial cells. The cuticle forms taenidial folds projecting into the lumen, which help to prevent the collapse of the tracheal branches (58) (Figure 1C). The correct composition of the cuticle is essential for tracheal development and the function of epithelia (73). Recently, it was shown that the scavenger receptor class B Debris buster (*Dsb*) is a key player for sorting critical components of the cuticle enabling airway functionality (74). The airways of *Dsb* mutants were strongly affected, especially the dorsal trunk elongation was disrupted, finally even leading to breaks in the dorsal trunk of older larvae (74). Unsurprisingly, those larvae died from hypoxia. This is just one example, underlying the importance of the integrity of the cuticle for the proper functioning of the epithelium.

Since the branching pattern of the tracheal system during development and larval life is highly conserved, the integrity or remodeling of it can be used as an indicator for the molecular role of certain risk genes for airway diseases during development, or to assess the influence of air-born stressors. For example, it could be shown that animals with overexpression of the asthma risk gene Orosomucoid-like (*ORMDL*) in the airways developed significantly fewer and shorter terminal branches, than control animals, leading to a reduced respiratory surface (75). These animals reacted strongly to the confrontation with hypoxia, and nuclear translocation of *sima* could be studied even in normoxia (75), indicating that terminal sprouting mechanism (71) might have been activated in these animals. Transcriptome analysis of the tracheae of *ORMDL* knock-down and overexpression animals revealed downregulation of several components of the epidermal growth factor receptor (EGFR) pathway, which is not only associated with a compromised epithelial barrier during severe asthma (32) but also crucial for the fate of the airway epithelium (64). In order to investigate the influence of e-cigarette vapor on the offspring, adult flies were exposed to e-nicotine (76). The offspring of these flies showed morphological changes of the trachea, which were significantly reduced in length. In a smoke induced *Drosophila*-COPD model, structural changes of the airway system, induced by cigarette smoke exposure, were characterized (77). The animals had a reduced respiratory surface, caused by the reduction of secondary and tertiary branches in length and number. Moreover, RNASeq analysis of isolated airway epithelia revealed regulation of genes involved in the response to xenobiotics and reactive oxygen species (ROS), as well as glutathione metabolism. Other signaling pathways were associated with stress responses and repair mechanisms. All noticeable altered expressed genes were related to COPD and disease progression, underlining *Drosophila* as a valuable model to study chronic lung diseases (77).

These examples underline that the characterization of structural and molecular changes in the airway epithelium of *Drosophila* can perfectly be used to identify and investigate the effects of key players in respiratory disease development. In addition, the simple structure of the airways with its single-layered epithelium allows studying the immunologic mechanisms of respiratory epithelium, which are often involved in respiratory disease development. It has to be kept in mind that, despite this seemingly very simple organization the airway epithelium of flies has to cope with exactly the same problems and challenges as is the case for the human airway epithelium. In the latter case, these different tasks are performed by different cell types. There are first indications that the complexity of the cells of the *Drosophila* airway epithelium is far greater than assumed earlier (78) and that, in particular, tasks such as mucin secretion can also be performed by normal airway epithelial cells in *Drosophila*. It should also be noted that the biology of airway epithelial precursor cells is excellently reproduced in the *Drosophila* system (79). It follows that the functional performance of the *Drosophila* airway epithelium is not too far from that of the human epithelium and that these performances are, however, partly provided by different cell types.

## Organization of the insect tracheal immune system

Respiratory organs, whether they are the lungs of vertebrates or tracheae of insects, serve as ports of entry for microbes (80–82). Their large surface area is covered by a very thin epithelial layer, both of which are necessary for effective gas exchange and as such represent a more or less fixed structure. Therefore, respiratory systems had to develop very efficient local immune responses to prevent penetration of the fragile epithelial barrier by viruses, bacteria, fungi, or other parasites. Innate epithelial immune responses accordingly play a central role, particularly in invertebrates and basal vertebrates that rely solely on such systems. The response to bacteria that invade airway systems is more complex than previously anticipated because the host microbiota in healthy lungs must be tolerated by the host immune system (83, 84); however, for insects, it is unknown up to now whether the tracheal system of insects harbors an indigenous microbiota.

**Figure 2:**
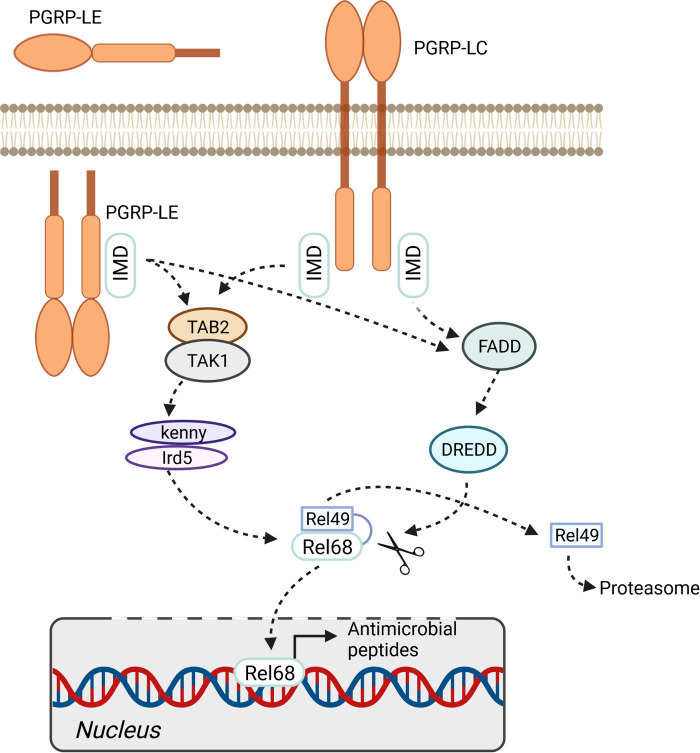
IMD pathway. The immune deficiency pathway can be activated by membrane bound pattern recognition receptor *PGRP-LC* or soluble intra- and extracellular receptor *PGRP-LE*. The activation of the pathway finally leads to the phosphorylation and cleavage of the transcription factor relish (Rel68/Rel49). The expression of antimicrobial peptides is activated by translocation of Rel68 into the nucleus. Figure was created with BioRender.com.

In insects, epithelial and systemic innate immunity function differently. The systemic immune system recognizes pathogen-associated patterns (PAMPs) *via* two parallel signaling pathways: the Toll-like receptor and immune deficiency (IMD) pathways. Although both signaling pathways activate NF-*κ*B factors, in epithelial tissues, Toll-signaling is usually not operative such that epithelial immunity mainly depends on the IMD pathway (85–88). In contrast to systemic immunity, the innate immune system of the epithelium is multifaceted and includes both physical and chemical barriers. To create an efficient physical barrier, major tasks are performed by different components working together. In the vertebrate respiratory system these include (i) effective filtration systems that prevent inhalation of larger particles; (ii) a sophisticated mucus layer that intercepts inhaled microorganisms; and (iii) effective anterograde transport of this mucus layer to remove trapped particles and microorganisms (89). Comparable mechanisms are operative in the insect tracheal system. Presumably, the following are of greatest relevance with respect to physical barrier function: (i) a sophisticated filter system that prevents entry of particles into the tracheal system (90); and (ii) an inner chitinous layer that protects airway epithelial cells to ensure the physical stability of the entire system (90, 91). It is not known whether the insect tracheal system possesses an anterograde transport system that enables the removal of inhaled particles. Thus, most potential airborne infections are likely to be prevented by these exquisite physical barriers within the tracheal system. Once a microorganism manages to enter the tracheal system, the next step is invasion through the epithelial barrier; this process triggers an effective, locally acting immune response (92, 93) which is mainly based on antimicrobial peptides (AMPs) that are produced and released locally by immunocompetent airway epithelial cells. As mentioned earlier, this highly potent antimicrobial response is mediated mainly *via* the IMD pathway (55, 92, 93) (Figure 2).

The most important PAMP sensors that operate in the airway epithelium are the peptidoglycan recognition receptors PGRP-LC and PGRP-LE, which interact with IMD to activate the NF-*κ*B factor Relish. PGRP-LC recognizes diaminopimelic acid-type peptidoglycans (DAP-type) at the membrane, whereas PGRP-LE responds to smaller pattern molecules that occur intracellularly. Moreover, an additional membrane-associated PGRP (PGRP-LA) influences the local immune response as a positive regulator of the IMD pathway (94). Activation of Relish, which occurs in response to activation of the IMD pathway, launches a highly potent antimicrobial response that is mediated primarily by AMP-encoding genes (Figure 2). Although canonical Toll signaling is not operative in the insect trachea, two Toll receptor genes, 18-wheeler and Tollo, are present at high levels in this tissue (55). Tollo does not form part of the antimicrobial response system in the trachea, although surprisingly, activation of this signaling system inhibits the IMD pathway (95). Moreover, Tollo couples not to MyD88 but to Ect4/SARM, thereby interfering with IMD-pathway signaling. Apparently, Tollo-dependent inhibition of IMD signaling is required to dampen the antimicrobial signature induced in response to IMD-pathway activation. If not regulated by this inhibitory input, activation of IMD might cause tissue damage due to unhindered immune effector activity. It is not clear whether this amounts to constitutive inhibition of epithelial immunity, or whether it is a tightly regulated brake (95).

In summary, it can be concluded that the epithelial immune system of the respiratory tract of *Drosophila* is a phylogenetically primordial module that is active in a similar form in the epithelia of mammals and thus also in the epithelia of humans. The explicit omission of adaptive immunity is given and cannot be disregarded. However, this apparent deficiency opens up a direct view of epithelial immunity and its importance for the orchestration of pathological processes without being obscured by complex feedback systems and mutual influences.

## Immune activity induced airway remodeling

In murine models as well as in human asthma patients, NF-*κ*B dependent processes occurring in airway epithelial cells have been identified to be involved in airway remodeling processes (96, 97). These studies led to the general idea that epithelial NF-*κ*B is a hub that connects immune responses with airway remodeling (98, 99). Recently, an immune-induced remodeling phenotype of the airway was characterized in *Drosophila* (100). Here, activation of the IMD-pathway, which is the equivalent of the TNF-α-pathway, led to substantial airway remodeling comprising thickening of the airway epithelium, irregular cell-cell junctions, and impairments of the air-conducting lumen. A detailed analysis revealed that these remodeling phenotypes could unexpectedly be attributed to NF-*κ*B activation to a relatively small proportion. Instead, an alternative signaling pathway is operative. Here, IMD-pathway signaling at the level of Tak1 realizes a branch to the c-Jun N-terminal kinases (JNK) pathway, which then leads to its activation and which is also responsible for the vast majority of the structural remodeling of the *Drosophila* airways in response to epithelial immune activation (100). This dual role of Tak1 had been described as a physical link between immune and stress signaling pathways (101). Moreover, activation of the JNK pathway is also known as a mechanism to induce remodeling processes in chronic airway disease models (102, 103). Thus, we see similar mechanisms between different lung disease models with respect to the mechanisms underlying remodeling processes. In *Drosophila* it was shown that activation of the epithelial JNK pathway, in turn, leads to the activation of the transcription factor Forkhead box O (FoxO), which is responsible for the observed structural changes of the airway epithelium (100). In this way, this important immune signaling pathway of airway epithelia controls both the activation of classical immune responses and the processes leading to structural changes. However, the latter only occurs in the case of chronic, long-lasting activation of these signaling pathways and thus represents a reaction to being classified as pathological, which can also be observed in various chronic diseases of the airways (104). FoxO factors are of particular interest in this context because, in addition to coordinating these structural changes, they themselves show an important function in the context of the immune response, which has been shown for different epithelia (105–107). Moreover, FoxO factors have recently been shown to be of central importance for the pathogenesis of different chronic lung diseases such as idiopathic pulmonary fibrosis and pulmonary hypertension (108, 109). Thus, the *Drosophila* model of immune- and stress-induced airway remodeling connects the different studies on remodeling processes, as it delivers a comprehensive understanding of the underlying molecular mechanisms.

## Limitations of the model

Although we are fully convinced of the many outstanding benefits of the *Drosophila* model for the analysis of airway remodeling, the limitations of this model should also always be considered. First of all, one always has to keep in mind that flies are not humans, but the same applies to the statement that mice are not humans. This means that there are always differences between animal models and the patient situation. In the case of *Drosophila*, it is (1) the lack of adaptive immunity, (2) the much simpler structured airway epithelium, as well as (3) the also much simpler subepithelial structures, which must be particularly considered here. On the one hand, this means that especially those processes that depend on adaptive immunity, that are purely directed to cell subtypes of the airway epithelium and that focus on the role of subepithelial structures are not optimally represented in the Drosophila system. It should be noted, however, that the anticipated simplicity of the Drosophila airway epithelium is probably not that simple and that processes such as secretion of mucins could very well be studied in this system. However, if one is aware of these limitations, the full strength of the model unfolds, in which indeed the outstanding importance of the epithelium can be focused on.

## Future directions

Understanding the developmental pathways and immunological processes involved in airway remodeling is essential for the development of new therapeutic and/or preventive approaches. For obvious ethical reasons, it is extremely difficult to study early insults in infant lung tissue. In addition, the long life-span of humans makes the follow-up of such events very challenging. Even in mice it is demanding to study the effects of early insults on lung and organismal performance later in life. Therefore, *D. melanogaster*, because of its comparatively short life-cycle of approximately 2 weeks and a maximal lifespan of 2–3 months, provides a powerful tool to study the early stages of airway remodeling and its consequences for the development of new therapeutic and/or preventive approaches. On the more, about 75% of human disease genes have homologs in *D. melanogaster* (110).

Thus, the highly conserved epithelial signaling pathways identified in the fly can be prioritized for further mechanistic studies in the interplay with more complex cell assemblies of the airways of mammalian models. In parallel, such pathways can be tested in human culture systems with progressively higher levels of complexity, providing information to what extent these findings are transferable to humans.

**Figure 3:**
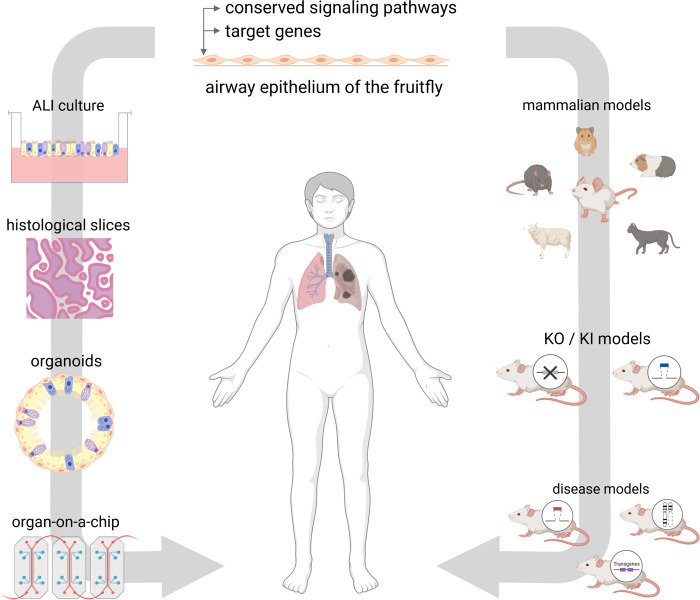
Future directions. The airway epithelium of *D. melanogaster* offers a great tool for the investment of conserved pathways and target genes. On that basis, findings can be investigated in mammalian models, such as knock-down (KD) and knock-in (KI) models or disease models. On the other hand, findings can be transferred to *in vitro* models using human cell culture systems, histological slices, organoids or organ-on-a-chip approaches. Figure was created with BioRender.com.

These culture systems range from simple air-liquid interface cultures that, unlike flies, reflect the full spectrum of epithelial cell types, to precision-cut lung slides that additionally exhibit subepithelial structures, to organoid cultures, and finally to lung-on-a-chip that represent the full physiology of the airway including ventilation and perfusion (Figure 3).

At the same time the fly is an established tool for screening large substance libraries to identify novel drugs and treatment opportunities, which will be validated as described above before moving to human clinical trials and ultimately hopefully benefit for patients suffering from airway remodeling.
